# A multivariable Mendelian randomization analysis investigating smoking and alcohol consumption in oral and oropharyngeal cancer

**DOI:** 10.1038/s41467-020-19822-6

**Published:** 2020-11-27

**Authors:** Mark Gormley, Tom Dudding, Eleanor Sanderson, Richard M. Martin, Steven Thomas, Jessica Tyrrell, Andrew R. Ness, Paul Brennan, Marcus Munafò, Miranda Pring, Stefania Boccia, Andrew F. Olshan, Brenda Diergaarde, Rayjean J. Hung, Geoffrey Liu, George Davey Smith, Rebecca C. Richmond

**Affiliations:** 1 MRC Integrative Epidemiology Unit, Population Health Sciences, Bristol Medical School, University of Bristol, Bristol, BS8 1TL UK; 2 Bristol Dental Hospital and School, University of Bristol, Bristol, BS1 2LY UK; 3 Department of Population Health Sciences, Bristol Medical School, University of Bristol, Bristol, BS8 1TL UK; 4 University Hospitals Bristol and Weston NHS Foundation Trust National Institute for Health Research Bristol Biomedical Research Centre, University of Bristol, Bristol, BS1 3NU UK; 5 RD&E Hospital, University of Exeter Medical School, RILD Building, Exeter, UK; 6 Genetic Epidemiology Group, World Health Organization, International Agency for Research on Cancer, Lyon, France; 7 School of Psychological Science, Faculty of Life Sciences, University of Bristol, Bristol, BS8 1TL UK; 8 Section of Hygiene, University Department of Life Sciences and Public Health, Università Cattolica del Sacro Cuore, Roma, Italia; 9 Department of Woman and Child Health and Public Health-Public Health Area, Fondazione Policlinico Universitario A. Gemelli IRCCS, Roma, Italy; 10 Department of Epidemiology, Gillings School of Global Public Health, University of North Carolina, Chapel Hill, NC 27599 USA; 11 Department of Human Genetics, Graduate School of Public Health, University of Pittsburgh, and UPMC Hillman Cancer Center, Pittsburgh, PA 15260 USA; 12 Prosserman Centre for Population Health Research, Lunenfeld-Tanenbaum Research Institute, Sinai Health System, Toronto, Canada; 13 Dalla Lana School of Public Health, University of Toronto, Toronto, Canada; 14 Princess Margaret Cancer Centre, Toronto, Canada

**Keywords:** Cancer prevention, Head and neck cancer, Oral cancer, Computational biology and bioinformatics

## Abstract

The independent effects of smoking and alcohol in head and neck cancer are not clear, given the strong association between these risk factors. Their apparent synergistic effect reported in previous observational studies may also underestimate independent effects. Here we report multivariable Mendelian randomization performed in a two-sample approach using summary data on 6,034 oral/oropharyngeal cases and 6,585 controls from a recent genome-wide association study. Our results demonstrate strong evidence for an independent causal effect of smoking on oral/oropharyngeal cancer (IVW OR 2.6, 95% CI = 1.7, 3.9 per standard deviation increase in lifetime smoking behaviour) and an independent causal effect of alcohol consumption when controlling for smoking (IVW OR 2.1, 95% CI = 1.1, 3.8 per standard deviation increase in drinks consumed per week). This suggests the possibility that the causal effect of alcohol may have been underestimated. However, the extent to which alcohol is modified by smoking requires further investigation.

## Abstract (web summary)

Alcohol and smoking are both associated with oral/oropharyngeal cancer risk, but independent effects are unclear given their relatedness. Here, the authors use multivariable Mendelian randomization to show that both alcohol and smoking are independently causal for oral/oropharyngeal cancer.

## Introduction

Head and neck squamous cell carcinoma (HNSCC), which includes cancers of the oral cavity and oropharynx, is the world’s 6th most common cancer^1^. Prognosis remains poor, with survival ranging between 19 and 59% at 10 years^2^. Established risk factors include cigarette smoking and alcohol intake, as well as the human papilloma virus (HPV), which is mainly linked with oropharyngeal cases and thought to be sexually transmitted^3–5^. While a large proportion of cases of head and neck cancer are attributable to the combination of smoking and alcohol, the respective contribution of these risk factors is not clear given the strong association between these behaviours. Better estimation of the known risk effects may help identify or clarify the importance of other potential risk factors. One large pooled analysis^6^ from 17 European and American case-control studies (11,221 cases and 16,168 controls) found that, of the population attributable risk of smoking and alcohol, 4% could be attributed to alcohol alone and 33% to tobacco alone, with 35% explained by a greater than multiplicative joint effect of alcohol and tobacco combined for oral and oropharyngeal cancer. However, the apparent synergistic effect seen in observational studies may underestimate the independent effects of smoking and alcohol.

Mendelian randomisation (MR) is an approach which attempts to minimise issues of measurement error, reverse causation and confounding by using genetic variants which are randomly distributed at birth and are known to be reliably associated with modifiable risk factors of interest, to obtain causal effect estimates for these risk factors on disease outcomes^7,8^. To thoroughly evaluate the causal effects of both alcohol consumption and smoking on the risk of oral and oropharyngeal cancer, we first conducted univariable MR analysis. A recent genome-wide association study (GWAS) reported a genetic correlation (rg ~0.34, 95% CI = 0.3, 0.4, *p* = 6.7 × 10^−63^) between alcohol use and smoking initiation, suggesting that sequence variations overlap substantially and that there is a causal pathway operating between them^9^. To account for this correlation between smoking and alcohol, and to simultaneously investigate the independent effects of each, we conducted multivariable Mendelian randomisation^10,11^. As well as investigating the effects of smoking initiation, we aimed to capture a quantitative lifetime measure of smoking behaviour using the comprehensive smoking index (CSI). This was used to compare the independent causal effect of smoking with the continuous measure of alcoholic drinks per week^12^. Finally, given evidence of strong genetic correlation for both smoking and alcohol intake with other risky behaviours^13^, and the established link between sexual behaviour and HPV-driven head and neck cancer^14^, we conducted additional MR analysis of both risk tolerance and lifetime number of sexual partners on oral cavity (OC) and oropharyngeal cancer (OPC).

In this work we use multivariable MR to demonstrate strong evidence for an independent causal effect of both smoking and alcohol consumption on oral and oropharyngeal cancer. This suggests the possibility that the effect of alcohol may have been previously underestimated. However, the extent to which alcohol is modified by smoking requires further investigation.

## Results

### Univariable Mendelian randomisation

Using 176 single nucleotide polymorphisms (SNPs) robustly and independently associated with smoking initiation (Supplementary Data 1), univariable MR provided strong evidence that smoking increases risk of oral/ oropharyngeal cancer (IVW Odds Ratio (OR) 2.5, 95% CI = 1.6, 3.9, *p* = 6.94 × 10^−5^ per log odds of smoking) (Table 1, Supplementary Figs. 1, 2). The direction of effect was consistent across the four MR methods tested (IVW, weighted median, weighted mode and MR-Egger), although MR-Egger results had wider confidence intervals (CI) and were less reliable due to low *I*^2^ values of the SNP-exposure associations, indicating possible violation of the no measurement error (NOME) assumption (Supplementary Table 1). There was some evidence of heterogeneity in the SNP effects (Supplementary Table 2) although no SNP effect outliers were detected from MR-PRESSO and MR-Egger intercepts indicated limited evidence of directional pleiotropy (Supplementary Table 3). Effects were consistent, and ORs slightly larger, when the summary statistics for smoking initiation were obtained from a GWAS meta-analysis in GSCAN which excluded 23andMe and UK Biobank (IVW OR 3.3, 95% CI = 1.8, 6.1) and a GWAS conducted in the UK Biobank only (IVW OR 5.2, 95% CI = 1.8, 14.9) (Table 1).

**Table 1:** Univariable Mendelian randomisation of smoking and risk of oral and oropharyngeal cancer.

Exposure	Exposure dataset	Exposure *N*	*N* SNPs	F-stat	Method	OR	95% CI	*P*
Smoking initiation†	GSCAN	1,232,091	176	39.9	IVW*	2.50	1.59, 3.91	6.94E−5
					Weighted median	2.72	1.46, 5.05	0.001
					Weighted mode	6.84	1.00, 46.78	0.052
					MR-Egger**	5.34	0.47, 58.7	0.181
					MR-PRESSO	NA	NA	NA
Smoking initiation†	GSCAN without UK Biobank***	249,171	176	8.71	IVW*	3.33	1.81, 6.13	1.16E−4
					Weighted median	2.52	1.08, 5.85	0.032
					Weighted mode	3.38	0.54, 21.1	0.195
					MR-Egger**	4.8	0.57, 40.0	0.149
					MR-PRESSO	NA	NA	NA
Smoking initiation†	UK Biobank	461,066	176	18.4	IVW*	5.20	1.80, 14.9	0.002
					Weighted median	2.62	0.58, 11.9	0.213
					Weighted mode	0.77	0.06, 10.1	0.590
					MR-Egger**	0.37	0.01, 10.7	0.563
					MR-PRESSO	NA	NA	NA
Comprehensive smoking index‡	UK Biobank	462,690	108	45.2	IVW*	3.47	2.39, 5.03	5.97E−11
					Weighted median	3.45	1.96, 6.08	1.84E-5
					Weighted mode	3.38	0.87, 13.4	0.085
					MR-Egger**	3.16	0.40, 24.9	0.279
					MR-PRESSO	NA	NA	NA

All statistical tests were two-sided.

*F-stat* mean F-statistic, *IVW* inverse variance weighted, *OR* odds ratio, *CI* confidence intervals, *P p-value*, *NA* if no outliers detected.

*Random effects model.

**SIMEX given low *I*^2^ value, should be interpreted with caution.

†per log odds of smoking initiation.

‡per SD increase in Comprehensive Smoking Index.

***Also excludes 23andMe study.

Findings of a causal effect of smoking initiation on oral and oropharyngeal cancer risk were further supported by MR analysis using 108 SNPs associated with a lifetime measure of smoking behaviour, the comprehensive smoking index (CSI) (Supplementary Data 1). Here, a 1 standard deviation (SD) increase in the CSI (equivalent to an individual smoking 20 cigarettes a day for 15 years and stopping 17 years ago, or an individual smoking 60 cigarettes a day for 13 years and stopping 22 years ago) was found to increase oral and oropharyngeal cancer risk combined, with an IVW OR of 3.5 (95% CI = 2.4, 5.0, *p* = 5.97 × 10^−11^) (Table 1, Supplementary Figs. 3, 4, Supplementary Tables 1–3).

**Fig. 1:**
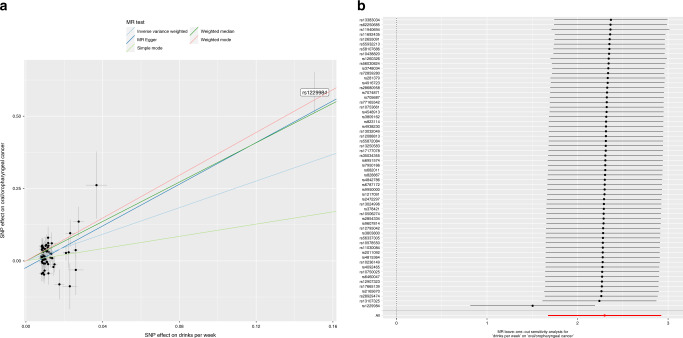
Scatter and leave one out plots demonstrating influential outliers in univariable MR of alcohol consumption and oral and oropharyngeal cancer risk. **a** Scatter plot showing *ADH1B* (rs1229984), an outlier single nucleotide polymorphism in the analysis of alcohol consumption (drinks per week, *n* = 941,280) and oral and oropharyngeal cancer risk (*n* = 6034 cases and 6585 controls). **b** Leave one out plot again demonstrating the *ADH1B* (rs1229984) influential outlier. Consistent evidence for a causal effect of alcohol on oral and oropharyngeal cancer risk was found even when this variant was excluded from the analysis. Effect estimates are reported per SD increase in the exposure and error bars represent 95% confidence intervals.

Using 60 SNPs robustly and independently associated with number of alcoholic drinks per week, univariable MR provided strong evidence that increased alcohol consumption increases risk of oral/oropharyngeal cancer. Here a 1 SD increase in drinks per week (equivalent to 9 additional drinks per week) was found to increase oral and oropharyngeal cancer risk combined, with an IVW OR of 10.0 (95% CI = 5.3, 18.6, *p* = 5.64 × 10^−13^) (Table 2). The direction of effect was consistent across the four MR methods tested and high *I*^2^ values of the SNP-exposure associations indicated low bias from regression dilution (i.e., attenuation towards the null of an association as a result of random measurement error) (Supplementary Table 1). However, there was some heterogeneity in the SNP effect estimates (Supplementary Table 2) and MR-Egger intercepts indicated some evidence for directional pleiotropy (Supplementary Table 3). Effects were consistent, although ORs slightly smaller, when the summary statistics for drinks per week were obtained from a GWAS meta-analysis in GSCAN which excluded 23andMe and UK Biobank (IVW OR 8.3, 95% CI = 4.7, 14.4) and a GWAS conducted in the UK Biobank only (IVW OR 5.8, 95% CI = 3.7, 9.0) (Table 1). In the main analysis, MR-PRESSO identified an influential outlier which was the *ADH1B* variant, rs1229984 (outlier test *p-*value = 0.024) which was also shown to be a clear outlier in both scatter and leave-one-out plots (Fig. 1). With removal of this outlier, the causal effect estimate was halved but still large (OR 4.5, 95% CI = 2.3, 9.0). The MR-Egger intercept indicated no further directional pleiotropy once this variant was removed (Supplementary Table 3).

**Table 2:** Univariable Mendelian randomisation of alcohol consumption and risk of oral and oropharyngeal cancer.

Exposure	Exposure dataset	Exposure *N*	*N* SNPs	F-stat	Method	OR	95% CI	*P*
Drinks per week☨	GSCAN	941,280	60	74.7	IVW*	9.96	5.33, 18.6	5.64E−13
					Weighted median	30.0	12.6, 71.5	1.47E−14
					Weighted mode	40.4	15.72, 103.8	1.88E−10
					MR-Egger	36.7	14.1, 95.5	6.80E−10
					MR-PRESSO	4.50	2.26, 8.96	7.12E−5
	GSCAN without UK Biobank***	226,223	60	17.1	IVW*	8.25	4.74, 14.4	8.04E−14
					Weighted median	19.4	98.9, 42.1	5.09E−14
					Weighted mode	17.1	7.69, 37.8	2.92E−9
					MR-Egger	16.4	7.47, 36.0	3.27E−9
					MR-PRESSO	NA	NA	NA
	UK Biobank	414,343	60	49.0	IVW*	5.80	3.70, 9.01	1.54E−14
					Weighted median	12.4	6.79, 22.7	3.04E−16
					Weighted mode	11.3	6.25, 20.4	5.09E−11
					MR-Egger	9.83	5.26, 18.4	1.55E−9
					MR-PRESSO	NA	NA	NA

All statistical tests were two-sided.

*F-stat* mean F-statistic, *IVW* inverse variance weighted; *OR* odds ratio, *CI* confidence intervals, *P p-value*, *NA* if no outliers detected.

*Random effects model.

☨per SD increase in drinks per week

***Also excludes 23andMe study.

### Stratification by cancer subsite

**Fig. 2:**
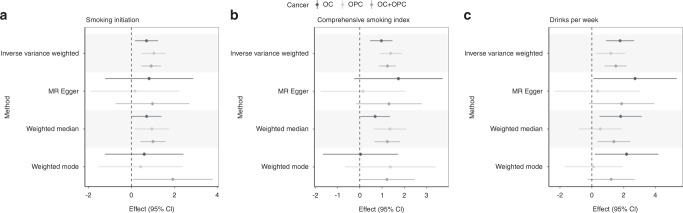
Univariable Mendelian randomisation of smoking initiation, lifetime smoke exposure and drinks per week on oral and oropharyngeal cancer subsites. Univariable effects displayed were obtained using summary-level data from the GWAS of (**a**) smoking initiation (*n* = 1,232,091), (**b**) comprehensive smoking index (*n* = 462,690) and (**c**) drinks per week (*n* = 941,280) on oral and oropharyngeal cancer risk (*n* = 6034 cases and 6585 controls). Smoking initiation estimates are reported per log odds increase, while comprehensive smoking index and drinks per week are reported per SD increase in drinks per week. Error bars represent 95% confidence intervals. All statistical tests were two-sided.

In MR analysis stratified by cancer subsite, we found evidence for a causal effect of smoking initiation in both oral cavity (IVW OR 2.0, 95% CI = 1.2, 3.4) and oropharyngeal cancer (IVW OR 2.8, 95% CI = 1.6, 4.9); comprehensive smoking index in OC (IVW 2.6, 95% CI = 1.6, 4.3) and OPC (IVW 4.0, 95% CI = 2.5, 6.5); and alcoholic drinks per week in OC (IVW OR 5.9, 95% CI 2.4, 14.2) and OPC (IVW OR 3.3, 95% CI = 1.3, 8.1), which were consistent across the MR methods (Fig. 2).

### Multivariable Mendelian randomisation

**Fig. 3:**
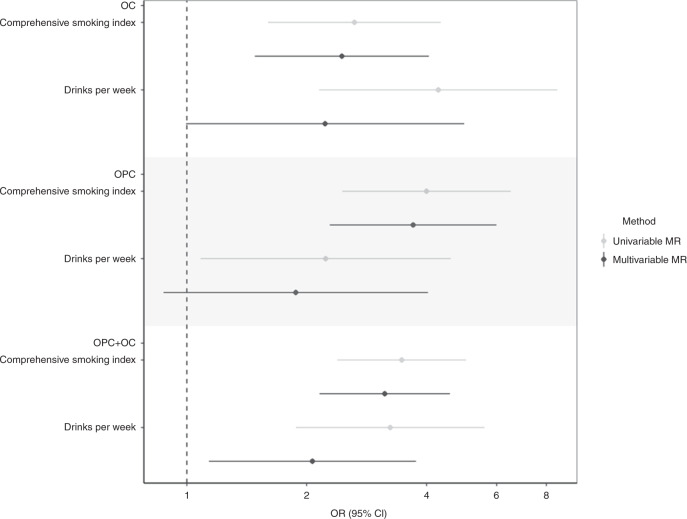
Multivariable Mendelian randomisation of lifetime smoke exposure and drinks per week on oral and oropharyngeal subsites. Effect estimates (ORs) are reported per SD increase in the exposure of drinks per week (*n* = 226,223) and the comprehensive smoking index (*n* = 226,223) on oral and oropharyngeal cancer risk (*n* = 6034 cases and 6585 controls). Error bars represent 95% confidence intervals. Genetic instrument for drinks per week excludes outlying variant, *ADH1B* (rs1229984). Univariable and multivariable effects displayed were obtained using summary-level data from the GWAS of comprehensive smoking index in UK Biobank and summary-level data from a GWAS of drinks per week in GSCAN, excluding UK Biobank and 23andMe. All statistical tests were two-sided.

In the multivariable MR analysis controlling for alcohol consumption, there was strong evidence for a direct causal effect of lifetime smoking behaviour on risk of oral/oropharyngeal cancer (IVW OR 2.6, 95% CI = 1.7, 3.9 per SD increase in the CSI) (Table 3). In multivariable MR analysis controlling for lifetime smoking, there was also strong evidence for a direct causal effect of alcohol consumption on risk of oral/oropharyngeal cancer (IVW OR 5.2, 95% CI = 3.2, 8.6 per SD increase in drinks per week) (Table 3). The independent causal effects estimated from multivariable MR-Egger were consistent with the IVW analysis for both alcohol consumption and lifetime smoking (Table 3). While there was limited evidence for heterogeneity in the SNP effect estimates indicating instrument validity, the MR-Egger intercept deviated from the null, which is suggestive of directional pleiotropy. When we removed the *ADH1B* variant found to be an outlier in the univariable MR analysis, the independent effects of both smoking and alcohol on risk of oral/oropharyngeal cancer persisted remained, although the magnitude of the effect was reduced for alcohol (IVW OR 2.1, 95% CI = 1.1, 3.8) (Table 3, Fig. 3). The MR-Egger intercept indicated no further directional pleiotropy once this variant was removed (Supplementary Table 3).

**Table 3:** Multivariable Mendelian randomisation for smoking initiation and alcohol consumption with risk of oral and oropharyngeal cancer.

Exposure	Exposure dataset	*N* SNPs	Q-stat for instrument strength	F-stat	Q-stat for instrument validity	*P*-value for instrument validity	Method	OR	95% CI	*P*
MR analysis using full set of independent SNPs for alcohol and smoking										
Comprehensive Smoking Index†	UK Biobank	108	5056	30.1	190.5	0.085	IVW	2.63	1.76, 3.90	1.67E−6
							MR-Egger	3.24	2.14, 4.92	3.24E−8
Drinks per week☨	GSCAN without UK Biobank*	60	1141	6.79			IVW	5.22	3.16, 8.64	1.16E−10
							MR-Egger	8.94	4.76, 16.8	8.94E−12
MR analysis without *ADH1B*										
Comprehensive Smoking Index†	UK Biobank	108	3117	18.7	170.9	0.340	IVW	3.14	2.15, 4.58	2.68E−9
							MR-Egger	3.17	2.13, 4.73	1.43E−8
Drinks per week☨	GSCAN without UK Biobank*	59	825	4.94			IVW	2.07	1.14, 3.76	0.017
							MR-Egger	2.18	0.87, 5.45	0.094

IVW and MR-Egger regression are two-sided statistical tests.

*IVW* inverse variance weighted, *OR* odds ratio, *CI* confidence intervals, *P p-value,* *Q-stat* Cochran’s Q statistic, *F-stat* conditional F-statistic, *also excludes 23andMe study.

†per SD increase in Comprehensive Smoking Index.

☨per SD increase in drinks per week.

In multivariable MR analysis stratified by cancer subsite, we found evidence for a causal independent effect of lifetime smoking in OC (IVW OR 2.5, 95% CI = 1.5, 4.1) and OPC (IVW 3.7, 95% CI = 2.3, 6.0); and for alcoholic drinks per week in OC (IVW OR 2.2, 95% CI = 1.0, 5.0) and OPC (IVW OR 1.9, 95% CI = 0.9, 4.0) (Fig. 3).

### Investigating other risky behaviours

**Fig. 4:**
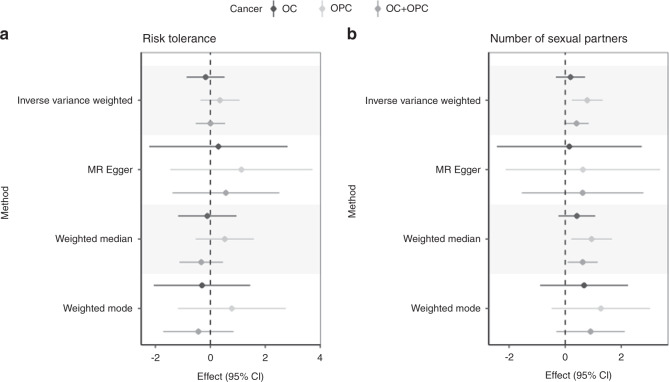
Univariable Mendelian randomisation of risk tolerance and number of sexual partners on oral and oropharyngeal cancer subsites. Effect estimates (log odds) are reported per SD increase in (**a**) risk tolerance (*n* = 508,782) and (**b**) number of sexual partners (*n* = 370,711). There was some evidence for a causal effect of number of sexual partners specific to the oropharyngeal subsite. Error bars represent 95% confidence intervals. All statistical tests were two-sided.

We found limited evidence to suggest there is a causal effect of risk tolerance more generally on risk of oral and oropharyngeal cancer (IVW OR 1.0, 95% CI = 0.6, 1.7 per SD) which was consistent across the MR methods applied (Fig. 4). There was some evidence for a causal effect of lifetime number of sexual partners (IVW OR 1.5, 95% CI = 1.0, 2.3 per SD), which was specific to oropharyngeal (IVW OR 2.2, 95% CI = 1.3, 3.8 per SD) rather than oral cavity cancer risk (IVW OR 1.2, 95% CI = 0.7, 2.0), and reasonably consistent across the MR methods applied (Fig. 4).

## Discussion

In this study, we applied both univariable and multivariable Mendelian Randomisation to determine a causal, independent effect of both smoking and alcohol consumption on the risk of oral and oropharyngeal cancer. The results were largely robust to sensitivity analyses accounting for horizontal pleiotropy. Effects were consistent between both oral cavity and oropharyngeal subsites and unlikely to be strongly influenced by risk tolerance and lifetime number of sexual partners.

Supporting of our findings regarding an independent effect of smoking, average relative risks (RR) of reported tobacco smoking have been found to range from 4.0–5.0 for both the oral cavity and oropharynx across 16 case-control and 3 cohort studies^15^. Alcohol use has been associated with an increased risk of oral and oropharyngeal cancer in a dose-dependent manner (RR 1.1 (95% CI = 1.0, 1.3) for light drinking to RR 5.1 (95% CI = 4.3, 6.1) for heavy drinking (>50 g alcohol per day)^16^. Hashibe et al^6^. investigated the independent effects of smoking and alcohol and found an OR of 2.4 (95% CI = 1.7, 3.4) for ever tobacco use among never alcohol drinkers and a more than multiplicative joint effect of tobacco and alcohol, but no clear effect of ever alcohol use among never tobacco users (OR 1.1, 95% CI = 0.9, 1.3), suggesting that alcohol use on its own may not play an important role^6^. Limitations of observational studies include recall bias, differential measurement error, as well as potential residual confounding, for example by socio-economic position, HPV status^17^ and other lifestyle factors^18–20^.

The independent effect for alcohol in our study suggests the possibility that previous observational estimates for alcohol may have been underestimated, but these cannot be directly compared given the differences in the methodological approaches and interpretation of estimates. MR estimates may reflect the effects of lifelong alcohol exposure, in comparison to the short-term effects captured in observational studies. Furthermore, while multivariable MR can estimate the independent (and unconfounded) effect of alcohol on oral/oropharyngeal cancer risk, it cannot determine the extent to which this effect is modified by smoking status, i.e., is it observed among smokers only or the whole population. Further individual level data analysis is required to determine this.

Several mechanisms have been suggested to explain the observed associations between alcohol and risk of head and neck cancer, including contaminants present in alcoholic drinks (e.g., N-nitrosodiethylamine in beer)^21^, free radical damage and impairment of DNA repair capacity^22,23^. Ethanol is oxidised to acetaldehyde, which has a direct carcinogenic effect and moreover alcohol may act as a ‘solvent’ for tobacco carcinogens and the induction of carcinogen-metabolising enzymes^24,25^. Given their strong co-existence, the effects of smoking and alcohol are often difficult to separate out. However, in addition to the reported synergistic effect, previous observational data has highlighted an independent effect of both agents^26^, with our results supporting this.

Studies which have used genetic variation in alcohol-metabolising genes do give some support to our findings. For example, East Asians who are homozygous for the (*2*2) variant allele of *ALDH2* (aldehyde dehydrogenase 2) are unable to metabolise acetaldehyde, which deters them from drinking alcohol, whereas those who are heterozygous (*1*2) have a 6-fold higher blood acetaldehyde concentration post-alcohol consumption with respect to the wild type *1*1. The OR of HNSCC among individuals with *2*2 has been found to be 0.5 (95% CI = 0.3, 1.0) and 1.8 (95% CI = 1.2, 2.8) among those with *1*2, relative to *1*1 individuals. These findings support the theory that alcohol increases HNSCC risk given the protective effect of non-drinking among homozygous individuals, while also indicating the carcinogenic action of acetaldehyde^27^. While the frequency of the *2 variant of *ALDH2* in non-Eastern populations is low, a recent GWAS revealed variation at other sites in the genome which are linked with alcohol consumption^9^. An MR analysis using these multiple variants robustly associated with alcohol from this recent GWAS^9^, demonstrated a causal effect of alcohol intake on HNSCC (OR 3.9, 95% CI = 1.3, 11.2) in the UK Biobank, although there were only 856 HNSCC cases included in this cohort^28^. A more recent multivariable MR study in UK Biobank^29^ investigated smoking initiation and alcohol consumption with multiple cancers. This study demonstrated that smoking initiation increased odds of head and neck cancer when adjusted for alcohol (IVW OR 1.4, 95% CI = 1.1, 1.8, *p* = 0.002). There was also a strong positive effect of alcohol consumption on head and neck cancer risk when adjusted for smoking (IVW OR 1.9, 95% CI = 0.8, 4.6, *p* = 0.142), although with wider confidence intervals^29^.

The current study applied both univariable and multivariable MR methods, using the largest number of SNPs identified from the latest GWAS for both smoking^9,12^, alcohol consumption^9,13^ and head and neck cancer^30^, that could be identified in the literature. A series of pleiotropy-robust MR methods and outlier detection were applied to rigorously explore the possibility that findings were not biased as a result of pleiotropy. Comparability of smoking initiation and alcohol consumption is an issue given that alcohol consumption is closer to a measure of lifetime use, whereas smoking initiation captures both light and short-term smokers and heavy, long-term smokers. To account for this and to evaluate the causal effects of a quantitative measure of smoke exposure, we performed univariable and multivariable MR using genetic variants identified in relation to a CSI^12^.

The effect of smoking and alcohol on HNSCC risk is thought to be synergistic, with their combined use interacting in a multiplicative manner^31^. We were not able to investigate this here as MR interaction analysis relies on individual-level data which we did not have access to^32^. We were also unable to investigate the potential carcinogenic role of acetaldehyde, distinct from alcohol intake, as has been done previously using alcohol dehydrogenase genotypes^27,33^, as this again typically relies on individual-level data to assess gene-by-environment interactions. Our finding that the *ADH1B* variant, rs1229984 was a clear outlier which inflated causal estimates, indicates pleiotropy of this variant via its role in alcohol metabolism. However, we found consistent evidence for a causal effect of alcohol on oral and oropharyngeal cancer risk when this variant was excluded from the analysis.

While we used a large number of genetic variants to serve as stronger instruments for both smoking and alcohol, these variants have not all been well characterised and indeed some of the top loci have been previously associated with other risky behaviours^13^. Therefore, additional analyses to evaluate the causal effects of risk tolerance and lifetime number of sexual partners on oral/oropharyngeal cancer were conducted. While there was no strong evidence for a causal effect of risk tolerance more generally, an effect of lifetime number of sexual partners was observed, specific only to oropharyngeal cancer risk. This effect is likely mediated through HPV and thought to be sexually transmitted^4,5^. While further work is required to investigate this effect of HPV status among oropharyngeal sites, it is unlikely to have substantially biased the MR estimates for smoking and alcohol, since no effect of lifetime number of sexual partners was observed in relation to oral cavity cancer risk, whereas similar effect estimates were observed in relation to alcohol and smoking. Finally, as most participants in the GAME-ON network were of European or North American decent, with only 10.8% from South America, more work is required to determine if our results translate to other ancestry groups.

In conclusion, this study used both univariable and multivariable MR analyses to demonstrate an independent causal effect for both smoking and alcohol on oral and oropharyngeal cancer risk. In particular, we observed large effects for alcohol in our multivariable MR analysis, which suggests the possibility that previous observational estimates for alcohol may have been underestimated. However, these cannot be directly compared given the differences in the methodological approaches and interpretation of estimates. Further work using individual-level data could provide more robust evidence for a synergistic effect and the carcinogenic role of acetaldehyde. Findings from this study add to a growing body of evidence from MR studies surrounding the harmful effects of alcohol consumption^34^ and should be used to guide public health messages regarding the harms of even moderate drinking.

## Methods

Univariable and multivariable Mendelian randomisation were applied using summary-level genetic data from the GWAS and Sequencing Consortium of Alcohol and Nicotine use (GSCAN)^9^, the UK Biobank study^12,13^, and a GWAS of oral and oropharyngeal cancer conducted by the Genetic Associations and Mechanisms in Oncology (GAME-ON) Network^30^, in a two-sample MR framework^35^. Mendelian randomisation is an approach which uses genetic variants as instruments to obtain estimates for the causal effect of these risk factors on disease outcomes. Three assumptions must be satisfied to ensure an MR study is valid which include: (1) genetic variants should be robustly associated with the risk factor of interest (i.e., the relevance assumption), (2) there are no confounders of the genetic variants-outcome association (the independence assumption) and (3) the exclusion restriction assumption. For further detail on the terms used in this study please read the Mendelian randomisation dictionary^36^.

### Summary-level data from GSCAN and UK Biobank

Summary-level genome-wide association studies were obtained for alcohol consumption (drinks per week, *n* = 941,280) and smoking initiation (a binary phenotype indicating whether an individual had ever smoked regularly) (*n* = 1,232,091) from the GSCAN study^9^. The comprehensive smoking index (CSI) was derived by Wootton et al in the UK Biobank (*n* = 462,690)^12^. This included information on smoking duration, heaviness and cessation, which were combined into a lifetime smoking index along with a simulated half-life (τ) constant. Summary-level GWAS data were obtained for this phenotype in the UK Biobank^12^.

### Summary-level data from GAME-ON

GWAS was performed on 6,034 HNSCC cases and 6,585 controls from 12 studies which were part of the GAME-ON network^30^. Cancer cases comprised the following ICD-10 codes: oral cavity (C02.0–C02.9, C03.0–C03.9, C04.0–C04.9 and C05.0–C06.9) and oropharynx (C01.9, C02.4 and C09.0–C10.9). The study population included participants from Europe (45.3%), North America (43.9%) and South America (10.8%). Details of the studies included, as well as the genotyping and imputation performed are published^30,37^.

### Univariable Mendelian randomisation

To assess causal effects of smoking and alcohol consumption, we identified 60 single nucleotide polymorphisms (SNPs) for alcohol consumption and 176 SNPs for smoking initiation reaching genome-wide significance (*p* < 5 × 10^−8^) in the GSCAN GWAS which were present in the GAME-ON GWAS (Supplementary Data 1, Supplementary Data 2). We also identified 108 independent SNPs associated with the CSI at *p* < 5 × 10^−8^ in the UK Biobank which were present in the GAME-ON GWAS (Supplementary Data 1, Supplementary Data 2).

Instrument strength was determined by the magnitude and precision of association of the genetic instruments with the risk factor, which was considered to be sufficient if the corresponding F-statistic is >10. Linkage disequilibrium statistics (*r*^2^) were evaluated to check the overlap between smoking initiation, comprehensive smoking index and drinks per week loci using LDmatrix. All pairwise correlations were low for the SNPs used to instrument smoking initiation and drinks per week (*r*^2^ < 0.06) and those used to instrument comprehensive smoking index and drinks per week (*r*^2^ < 0.07), with the exception of rs10236149 and rs6962772 which had an *r*^2^ of 0.743. For smoking initiation and comprehensive smoking index, there were 6 duplicate SNPs and 11 SNP pairs which were in strong LD (*r*^2^ > 0.8).

Two-sample MR analyses were conducted using the TwoSampleMR package (version 0.5.5) in R (version 3.5.3), to extract the SNPs instrumenting the risk factor from the oral/oropharyngeal cancer GWAS^38^. We next performed harmonisation of the direction of effects between exposure and outcome associations, where palindromic SNPs were aligned when minor allele frequencies (MAFs) were less than 0.3 or were otherwise excluded. SNP-specific Wald estimates were calculated (SNP-outcome estimate divided by SNP-exposure estimate) and an inverse variance weighted (IVW) random effects method applied to meta-analyse these to obtain an estimate for the causal effect of the risk factor on oral/oropharyngeal cancer risk.

To further assess the robustness of findings, we inspected the Cochran’s Q statistic which assesses heterogeneity between individual genetic variants, and which may indicate the presence of invalid instruments (e.g., due to horizontal pleiotropy). Horizontal pleiotropy occurs when the genetic instruments are associated with more than one independent biological pathway, which can result in violation of the MR exclusion restriction assumption (i.e., the variable is not related to the outcome other than via the risk factor of interest). First-order inverse-variance weights were used to calculate both the IVW estimate and Cochran’s Q. A random effects model was used in the presence of heterogeneity^39^. Scatter and leave-one-out plots were produced to evaluate influential outliers and MR-PRESSO (Mendelian Randomisation Pleiotropy RESidual Sum and Outlier) was used to detect and correct for potential outliers (*p* < 0.05)^40^. MR-PRESSO is a unified framework that evaluates genetic pleiotropy in a standard MR model. It attempts to perform outlier removal in order to re-estimate the original exposure-outcome relationship to reduce bias in MR estimates. The IVW method will provide an unbiased estimate in the absence of horizontal pleiotropy or when horizontal pleiotropy is balanced^41^. To account for pleiotropy, we compared results with three other MR methods, which each make different assumptions about this: MR-Egger^42^, weighted median^43^ and weighted mode^44^. MR-Egger can provide unbiased estimates even when all SNPs in an instrument violate the exclusion restriction assumption. Where there was evidence of violation of negligible measurement error (NOME), assessed based on the *I*^2^ statistic, MR-Egger was performed with simulation extrapolation (SIMEX) correction^45^. SIMEX relies on simulation to estimate or reduce bias due to measurement error, considering additional data sets with increasing measurement error variance. The weighted median stipulates that at least 50% of the weight in the analysis stems from variants that are valid instruments^43^, while the weighted mode requires that the largest subset of instruments which identify the same causal effect to be valid instruments^44^.

### Stratification by cancer subsite

We further performed MR analysis for alcohol and smoking with stratification by cancer subsite to evaluate potential heterogeneity in the causal effects. For this we used GWAS summary data on a subset of 2641 oropharyngeal cases and 2990 oral cavity cases from the 6034 HNSCC cases in the GAME-ON Network GWAS used in the main analysis and the 6585 common controls^30^. A total of 954 individuals with HNSCC were excluded from this analysis since these were cases of hypopharynx and overlapping cancers.

We also conducted further sensitivity analyses to estimate causal effects using summary-level data obtained from the GWAS of smoking initiation and drinks per week in GSCAN excluding UK Biobank and 23andMe, and summary-level data obtained from a GWAS of smoking initiation^38^ and drinks per week^13^ in the UK Biobank only.

### Multivariable Mendelian randomisation

We next conducted two-sample multivariable MR analysis which included SNPs which were genome-wide significant in either the GSCAN GWAS of alcohol consumption or the UK Biobank GWAS of comprehensive smoking index. After excluding SNPs with a pairwise *r*^2^ greater than 0.001, 168 independent SNPs were used in the analysis. To remove sample overlap between the GWAS for alcohol consumption and CSI in the analysis, we used summary-level data obtained from the GWAS of drinks per week in GSCAN, excluding UK Biobank and 23andMe (*n* = 226,223) and summary-level data obtained from the GWAS of CSI in the UK Biobank (*n* = 462,690). Both the IVW and MR-Egger framework have been extended to estimate causal effects in multivariable MR analysis^46,47^, which was conducted using both the MVMR (version 0.2.0) and Mendelian Randomization^48^ (version 0.5.0) packages in R. We used generalised versions of Cochran’s Q statistical tests to assess both instrument strength and validity in the two-sample summary data setting, where the covariance between the effects of the genetic variants on each exposure was fixed at zero by using non-overlapping samples for each exposure. This was performed using the MVMR package (version 0.2.0) in R.

### Investigating other risky behaviours

We used 124 SNPs associated with risk tolerance and 118 SNPs associated with lifetime number of sexual partners from a large GWAS meta-analysis^13^. We performed MR using the same univariable methods described in the main analysis and also evaluated the effects of these exposures stratified by cancer subsite.

### Statistics and reproducibility

All genome-wide association study (GWAS) data used in this study had been previously replicated in independent datasets (see respective GWAS studies)^9,12,30^. In our study, MG and RR independently repeated all univariable and multivariable Mendelian randomisation analyses once, both successfully obtaining the same conclusions.

### Reporting summary

Further information on research design is available in the Nature Research Reporting Summary linked to this article.

## Supplementary information

Supplementary Information

Reporting Summary

Peer Review File

Description of Additional Supplementary Files

Supplementary Data 1

Supplementary Data 2

## Data Availability

GWAS summary statistics for the SNPs used to estimate the causal effects of smoking initiation, lifetime smoking and alcoholic drinks per week in this study are presented in Supplementary Data 1 and 2. Full summary statistics for the GAME-ON GWAS have been deposited in dbGAP (OncoArray: Oral and Pharynx Cancer; study accession number: phs001202.v1.p1)^30^. Published data from this study can also be found in: Lesseur, C. et al. Genome-wide association analyses identify new susceptibility loci for oral cavity and pharyngeal cancer. Nat Genet. 48, 1544-1550 (2016)^30^. Smoking initiation and alcohol consumption data (GSCAN study) are published in: Liu, M. Z. et al. Association studies of up to 1.2 million individuals yield new insights into the genetic aetiology of tobacco and alcohol use. Nat Genet. 51, 237 (2019)^9^. Comprehensive smoking index (CSI) data is published in: Wootton, R. E. et al. Evidence for causal effects of lifetime smoking on risk for depression and schizophrenia: a Mendelian randomisation study. *Psychol Med*, 1–9 (2019)^12^. UK Biobank approval was given for this project (ID 40644**-**Investigating aetiology, associations and causality in diseases of the head and neck). A copy of the data generated in this this analysis is available at: https://github.com/rcrichmond/smoking_alcohol_headandneckcancer^49^

## Code availability

Two-sample MR analyses were conducted using the TwoSampleMR package (version 0.5.5) in R (version 3.5.3). Multivariable MR analysis was conducted using both the MVMR (version 0.2.0) and MendelianRandomization (version 0.5.0)^48^ packages in R. A copy of the code used in this this analysis is available at: https://github.com/rcrichmond/smoking_alcohol_headandneckcancer^49^
