# Supplementary material for: A multivariable Mendelian randomization analysis investigating smoking and alcohol consumption in oral and oropharyngeal cancer
Source: Nat Commun. 2020 Nov 27;11:6071. doi: 10.1038/s41467-020-19822-6 (PMC7695733; doi:10.1038/s41467-020-19822-6)
Supplement: Supplementary file 1 — Supplementary Information [file 41467_2020_19822_MOESM1_ESM.docx]

Supplementary Information

Supplementary Table 1 Assessing violation of the “NO Measurement Error” (NOME) assumption for instruments used in MR-Egger regression.

Supplementary Table 2 Assessing heterogeneity of single nucleotide polymorphism (SNP) effect estimates in inverse-variance weighted (IVW) and MR-Egger regression.

Supplementary Table 3 Assessing directional pleiotropy through MR-Egger intercept.

Supplementary Figure 1 Scatter plot demonstrating influential outliers in univariable MR analysis of smoking initiation and oral and oropharyngeal cancer risk.

Supplementary Figure 2 Leave one out plot demonstrating influential outliers in univariable MR analysis of smoking initiation and oral and oropharyngeal cancer risk.

Supplementary Figure 3 Scatter plot demonstrating influential outliers in univariable MR analysis of the comprehensive smoking index and oral and oropharyngeal cancer risk.

Supplementary Figure 4 Leave one out plot demonstrating influential outliers in univariable MR analysis of comprehensive smoking index and oral and oropharyngeal cancer.

Supplementary Table 1 Assessing violation of the “NO Measurement Error” (NOME) assumption for instruments used in MR-Egger regression.

| Exposure | Exposure dataset | Isq unweighted |
| --- | --- | --- |
| Smoking initiation | GSCAN | 0.63 |
| Comprehensive smoking index | UK Biobank | 0.67 |
| Drinks per week | GSCAN | 0.95 |

Abbreviations: Isq, I-squared function.

Supplementary Table 2 Assessing heterogeneity of single nucleotide polymorphism (SNP) effect estimates in inverse-variance weighted (IVW) and MR-Egger regression.

| Exposure | Exposure dataset | Q IVW | df | P | Q MR-Egger | df | *P* |
| --- | --- | --- | --- | --- | --- | --- | --- |
| Smoking initiation | GSCAN | 229.0 | 175 | 0.004 | 229.0 | 174 | 0.003 |
| Comprehensive smoking index | UK Biobank | 104.8 | 107 | 0.571 | 103.7 | 106 | 0.544 |
| Drinks per week | GSCAN | 86.0 | 59 | 0.012 | 78.9 | 58 | 0.035 |

Abbreviations: Q, Q-statistic; df, degrees of freedom; *P, p-value*.

IVW, inverse variance weighted and MR-Egger regression are two-sided statistical tests.

Supplementary Table 3 Assessing directional pleiotropy through MR-Egger intercept.

| Model | Exposure | Exposure dataset | *N* SNPs | Estimate | SE | CIL | CIU | *P* |
| --- | --- | --- | --- | --- | --- | --- | --- | --- |
| UVMR | Smoking initiation | GSCAN | 176 | -0.001 | 0.010 | -0.019 | 0.018 | 0.943 |
| UVMR | Comprehensive smoking index | UK Biobank | 108 | -0.001 | 0.008 | -0.016 | 0.015 | 0.925 |
| UVMR | Drinks per week | GSCAN | 60 | -0.017 | 0.007 | -0.031 | -0.002 | 0.022 |
| UVMR | Drinks per week (excluding ADH1B) | GSCAN | 59 | -0.005 | 0.012 | -0.030 | 0.020 | 0.715 |
| MVMR | Comprehensive smoking index  Drinks per week | UK Biobank  GSCAN | 108 | -0.006 | 0.002 | -0.011 | -0.001 | 0.005 |
| MVMR | Comprehensive smoking index  Drinks per week (excluding ADH1B) | UK Biobank  GSCAN | 107 | 0.000 | 0.003 | -0.006 | 0.005 | 0.609 |

Abbreviations: UVMR, univariable Mendelian randomization; MVMR, multivariable Mendelian randomization; SE, standard error; CIL, lower confidence interval; CIU, upper confidence interval; *P, p-value*.

MR-Egger regression is a two-sided statistical test.

Supplementary Figure 1 Scatter plot demonstrating influential outliers in univariable MR analysis of smoking initiation and oral and oropharyngeal cancer risk.


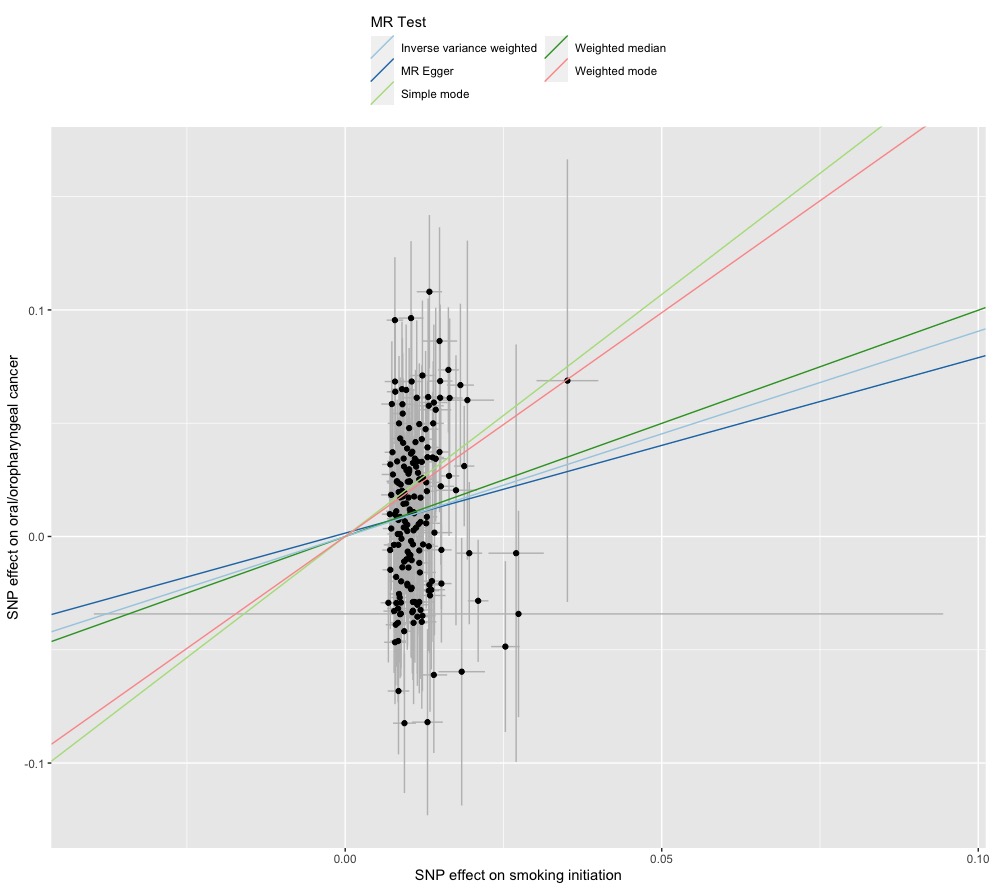


Scatter plot demonstrating no outliers in univariable MR analysis of smoking initiation (*n=* 1,232,091) on oral and oropharyngeal cancer risk (*n=* 6,034 cases and 6,585 controls). Effect estimates are reported per SD increase in the exposure and error bars represent 95% confidence intervals.


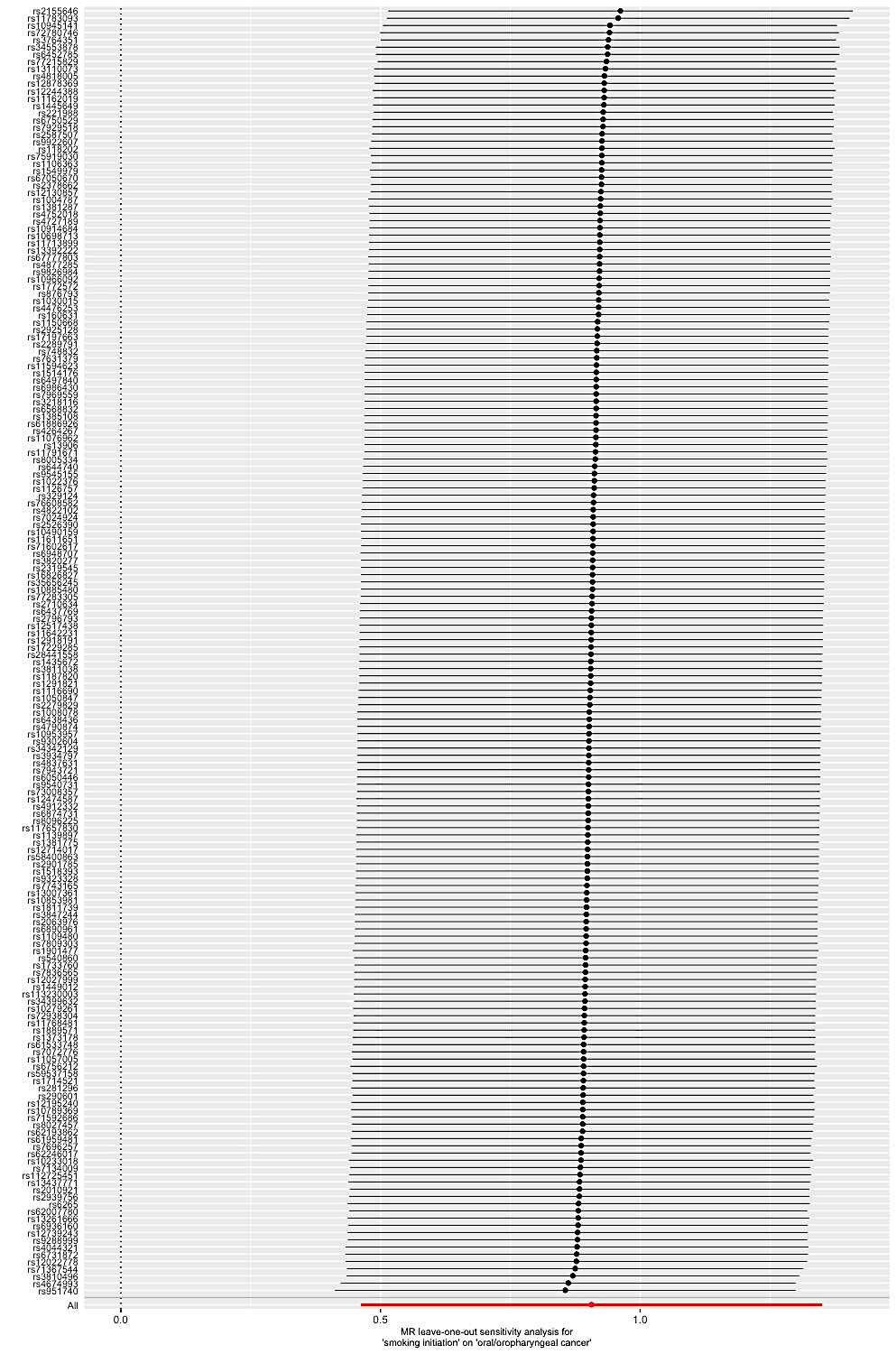
Supplementary Figure 2 Leave one out plot demonstrating influential outliers in univariable MR analysis of smoking initiation and oral and oropharyngeal cancer risk.

Leave one out plot demonstrating no outliers in univariable MR analysis of smoking initiation (*n=* 1,232,091) on oral and oropharyngeal cancer risk (*n=* 6,034 cases and 6,585 controls). Effect estimates are reported per SD increase in the exposure and error bars represent 95% confidence intervals.

Supplementary Figure 3 Scatter plot demonstrating influential outliers in univariable MR analysis of the comprehensive smoking index and oral and oropharyngeal cancer risk.


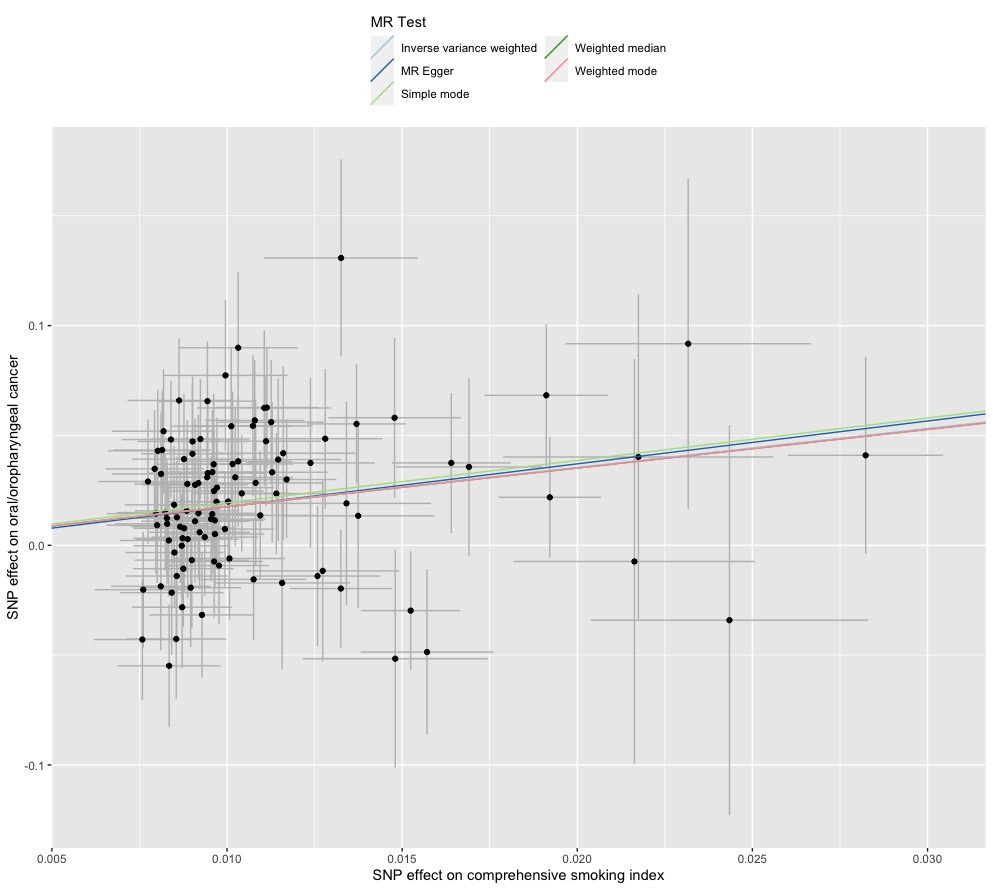


Scatter plot demonstrating no outliers in univariable MR analysis of comprehensive smoking index (*n=* 462,690) on oral and oropharyngeal cancer risk (*n=* 6,034 cases and 6,585 controls). Effect estimates are reported per SD increase in the exposure and error bars represent 95% confidence intervals.

Supplementary Figure 4 Leave one out plot demonstrating influential outliers in univariable MR analysis of comprehensive smoking index and oral and oropharyngeal cancer risk.


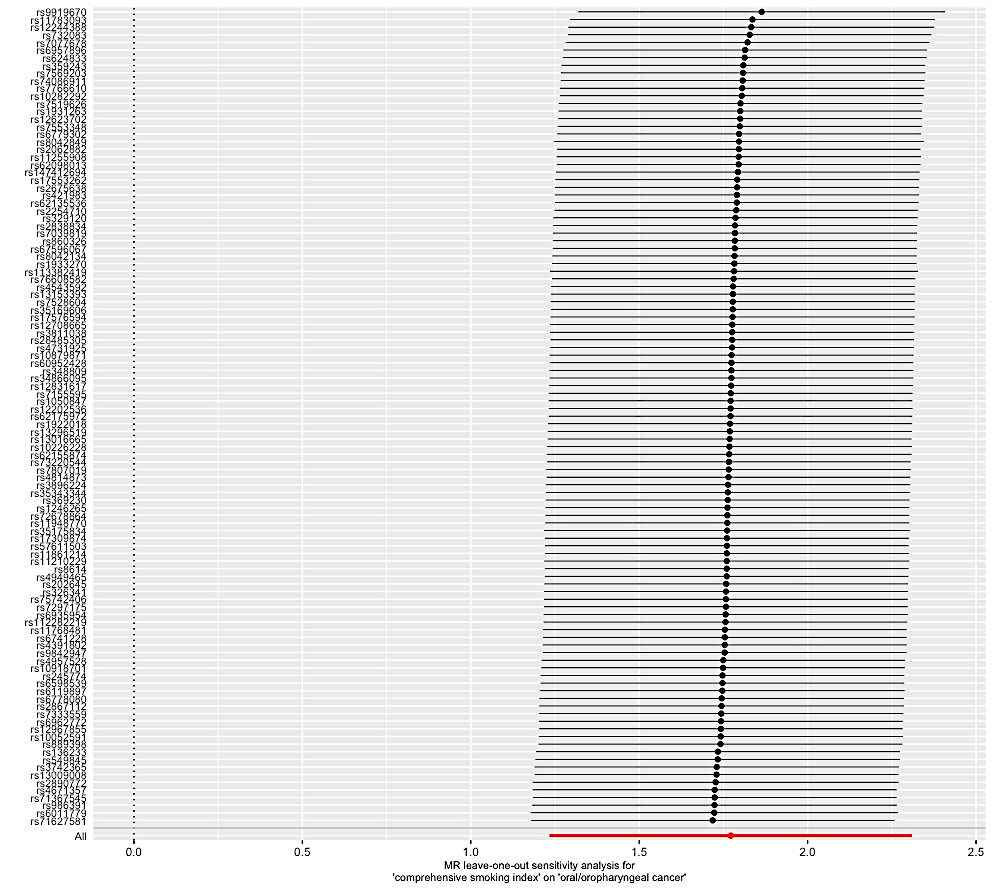


Leave one out plot demonstrating no outliers in univariable MR analysis of comprehensive smoking index (*n=* 462,690) on oral and oropharyngeal cancer risk (*n=* 6,034 cases and 6,585 controls). Effect estimates are reported per SD increase in the exposure and error bars represent 95% confidence intervals.
