# Supplementary material for: A multivariable Mendelian randomization analysis investigating smoking and alcohol consumption in oral and oropharyngeal cancer
Source: Nat Commun. 2020 Nov 27;11:6071. doi: 10.1038/s41467-020-19822-6 (PMC7695733; doi:10.1038/s41467-020-19822-6)
Supplement: Supplementary file 2 — Reporting Summary [file 41467_2020_19822_MOESM2_ESM.pdf]

nature research

Corresponding author(s): Mark Gormley

Last updated by author(s): Oct 13, 2020

Reporting Summary

Nature Research wishes to improve the reproducibility of the work that we publish. This form provides structure for consistency and transparency in reporting. For further information on Nature Research policies, see our Editorial Policies and the Editorial Policy Checklist.

Statistics

For all statistical analyses, confirm that the following items are present in the figure legend, table legend, main text, or Methods section.

n/a	Confirmed	
☐	☒	The exact sample size (*n*) for each experimental group/condition, given as a discrete number and unit of measurement
☐	☒	A statement on whether measurements were taken from distinct samples or whether the same sample was measured repeatedly
☐	☒	The statistical test(s) used AND whether they are one- or two-sided<br>*Only common tests should be described solely by name; describe more complex techniques in the Methods section.*
☐	☒	A description of all covariates tested
☐	☒	A description of any assumptions or corrections, such as tests of normality and adjustment for multiple comparisons
☐	☒	A full description of the statistical parameters including central tendency (e.g. means) or other basic estimates (e.g. regression coefficient) AND variation (e.g. standard deviation) or associated estimates of uncertainty (e.g. confidence intervals)
☐	☒	For null hypothesis testing, the test statistic (e.g. *F*, *t*, *r*) with confidence intervals, effect sizes, degrees of freedom and *P* value noted<br>*Give P values as exact values whenever suitable.*
☒	☐	For Bayesian analysis, information on the choice of priors and Markov chain Monte Carlo settings
☐	☒	For hierarchical and complex designs, identification of the appropriate level for tests and full reporting of outcomes
☐	☒	Estimates of effect sizes (e.g. Cohen's *d*, Pearson's *r*), indicating how they were calculated

Our web collection on statistics for biologists contains articles on many of the points above.

Software and code

Policy information about availability of computer code

Data collection: No software was used for data collection.

Data analysis: Code availability statement
Two-sample MR analyses were conducted using the TwoSampleMR package (version 0.5.5) in R (version 3.5.3). Multivariable MR analysis was conducted using both the MVMR (version 0.2.0) and MendelianRandomization (version 0.5.0) packages in R. A copy of the code used in this this analysis is available at: https://github.com/rcrichmond/smoking_alcohol_headandneckcancer

For manuscripts utilizing custom algorithms or software that are central to the research but not yet described in published literature, software must be made available to editors and reviewers. We strongly encourage code deposition in a community repository (e.g. GitHub). See the Nature Research guidelines for submitting code & software for further information.

Data

Policy information about availability of data

All manuscripts must include a data availability statement. This statement should provide the following information, where applicable:

- Accession codes, unique identifiers, or web links for publicly available datasets
- A list of figures that have associated raw data
- A description of any restrictions on data availability

GWAS summary statistics for the SNPs used to estimate the causal effects of smoking initiation, lifetime smoking and alcoholic drinks per week in this study are presented in Supplementary Data 1 and 2. Full summary statistics for the GAME-ON GWAS have been deposited in dbGAP (OncoArray: Oral and Pharynx Cancer; study accession number: phs001202.v1.p1). Published data from this study can also be found in: Lesseur, C. et al. Genome-wide association analyses identify new susceptibility loci for oral cavity and pharyngeal cancer. Nat Genet. 48, 1544-1550 (2016). Smoking initiation and alcohol consumption data (GSCAN study) are

published in: Liu, M. Z. et al. Association studies of up to 1.2 million individuals yield new insights into the genetic etiology of tobacco and alcohol use. Nat Genet. 51, 237 (2019). Comprehensive smoking index (CSI) data is published in: Wootton, R. E. et al. Evidence for causal effects of lifetime smoking on risk for depression and schizophrenia: a Mendelian randomisation study. Psychol Med, 1-9 (2019). UK Biobank approval was given for this project (ID 40644- Investigating aetiology, associations and causality in diseases of the head and neck). A copy of the data generated in this this analysis is available at: https://github.com/rcrichmond/smoking_alcohol_headandneckcancer

Field-specific reporting

Please select the one below that is the best fit for your research. If you are not sure, read the appropriate sections before making your selection.

[x] Life sciences [ ] Behavioural & social sciences [ ] Ecological, evolutionary & environmental sciences

For a reference copy of the document with all sections, see nature.com/documents/nr-reporting-summary-flat.pdf

Life sciences study design

All studies must disclose on these points even when the disclosure is negative.

Sample size

To enhance study study power, the largest available genome-wide association studies were used for both exposure and outcome measures to carry out univariable and multivariable Mendelian randomization. A number of studies were used which captured different smoking and alcohol behaviours, improving confidence in our results. Summary-level genetic data from the GWAS and Sequencing Consortium of Alcohol and Nicotine use (GSCAN), the UK Biobank study, and a GWAS of oral and oropharyngeal cancer conducted by the Genetic Associations and Mechanisms in Oncology (GAME-ON) Network, in a two-sample MR framework. Summary-level genome-wide association studies were obtained for alcohol consumption (drinks per week, n=941,280) and smoking initiation (a binary phenotype indicating whether an individual had ever smoked regularly, n=1,232,091) from the GSCAN study and the comprehensive smoking index (CSI) was derived by Wootton et al in the UK Biobank (n=462,690). For the disease outcome GWAS, 6,034 head and neck cancer cases and 6,585 controls derived from 12 studies which were part of the GAME-ON network.

Data exclusions

Pre-established exclusions included a total of 954 individuals with head and neck squamous carcinoma, since these were cases of hypopharynx and overlapping subsites which were therefore not representative of the oral or oropharyngeal cancer cases.

Replication

All genome-wide association study (GWAS) data used in this study had been previously replicated in independent datasets (see respective GWAS studies as described in the Data section). In our study, MG and RR independently repeated all univariable and multivariable Mendelian randomization analyses once, both successfully obtaining the same conclusions.

Randomization

Mendelian randomization is an approach which attempts to minimise issues of measurement error, reverse causation and confounding by using genetic variants which are randomly distributed at birth (during meiosis) and are known to be reliably associated with modifiable risk factors of interest, to obtain causal effect estimates for these risk factors on disease outcomes. With this in mind, Mendelian randomization has been compared to a natural randomized control trial, so in this study, those inheriting risk variants for alcohol consumption or smoking initiation were randomized to the case arm and those not inheriting risk variants in the control arm.

Blinding

In Mendelian randomization, estimation of the genetic instrument effect (beta), relies on randomization of the genotype during meiosis, which is similar to blinding to allocation (i.e. the instrument being robustly associated with exposure of interest and independent of unknown or unmeasured confounders) and the absence of any other pathway from the instrument to disease outcome. These are core assumptions for the method which were tested in our analysis.

Reporting for specific materials, systems and methods

We require information from authors about some types of materials, experimental systems and methods used in many studies. Here, indicate whether each material, system or method listed is relevant to your study. If you are not sure if a list item applies to your research, read the appropriate section before selecting a response.

Materials & experimental systems

n/a	Involved in the study	
[x]	[ ]	Antibodies
[x]	[ ]	Eukaryotic cell lines
[x]	[ ]	Palaeontology and archaeology
[x]	[ ]	Animals and other organisms
[x]	[ ]	Human research participants
[x]	[ ]	Clinical data
[x]	[ ]	Dual use research of concern

Methods

n/a	Involved in the study	
[x]	[ ]	ChIP-seq
[x]	[ ]	Flow cytometry
[x]	[ ]	MRI-based neuroimaging
