# Supplementary material for: A multivariable Mendelian randomization analysis investigating smoking and alcohol consumption in oral and oropharyngeal cancer
Source: Nat Commun. 2020 Nov 27;11:6071. doi: 10.1038/s41467-020-19822-6 (PMC7695733; doi:10.1038/s41467-020-19822-6)
Supplement: Supplementary file 4 — Description of Additional Supplementary Files [file 41467_2020_19822_MOESM4_ESM.pdf]

Description of Additional Supplementary Files

Supplementary Data 1
Single nucleotide polymorphisms (SNPs) robustly and independently related to smoking initiation, comprehensive smoking index and alcohol consumption

Supplementary Data 2
Summary-level data from GAME-ON
