# Supplementary material for: A multivariable Mendelian randomization analysis investigating smoking and alcohol consumption in oral and oropharyngeal cancer
Source: Nat Commun. 2020 Nov 27;11:6071. doi: 10.1038/s41467-020-19822-6 (PMC7695733; doi:10.1038/s41467-020-19822-6)
Supplement: Supplementary file 3 — Peer Review File [file 41467_2020_19822_MOESM3_ESM.pdf]

Reviewer #1 (Remarks to the Author):

Gormley et al present a comprehensive Mendelian Randomisation (MR) analysis to investigate the effect of smoking and alcohol consumption on oral and oropharyngeal cancer. They show in a univariable MR analysis that each exposure increases risk of oral and oropharyngeal cancer. In a multivariable MR analysis they demonstrate that the direct effects do not attenuate when accounting for the other exposure, suggesting that there are independent effects of both smoking and alcohol consumption.

The manuscript is well written and the MR analysis is well explained. My comments focus mainly on the MR analysis performed.

Major comments:

Main manuscript
1. Formalising smoking as exposure
Why did the authors select smoking initiation to formalise smoking exposure in the first instance? It is great to add the Comprehensive smoking index later, but why did the authors present this as a separate paragraph and not show this in the main analysis? How do the multivariable Mendelian Randomization results look like when using data from the Comprehensive smoking index?

2. Risky behaviour:
The authors also perform an univariable MR study where they look at the effect of other risky behaviours (risk tolerance and lifetime number of sexual partners) on the outcome. It would be nice to perform a multivariable MR study with both smoking, alcohol and risky behaviour to investigate attenuation when accounting for risky behaviour.

In the discussion the authors say: "unlikely to be strongly influenced by other risky behaviours" this needs to be rephrased as only two types of risky behaviours (risk tolerance and lifetime number of sexual partners) were actually investigated.

Methods:
3. "There was no direct overlap between the SNPs used to instrument alcohol consumption and smoking initiation."
What exactly do you mean by this? What do we learn from this? SNPs may be in LD ... Please clarify or delete this sentence.

4. To account for directional pleiotropy, we compared results with three other MR methods, which each make different assumptions about this: MR-Egger 14, weighted median 15 and weighted mode 16.

Do median and mode account for directional pleiotropy? Both methods are robust to general pleiotropy but NOT directional pleiotropy. You may want to rephrase: "To account for pleiotropy"

5. So far the manuscript only presents scatter and leave one out plots for alcohol as exposure. Please include into the Supplementary similar plots for all analysis (Smoking as exposure in univariable MR and multivariable MR)

6. The outlier selection for multivariable MR needs more details. How was the Q-statistic computed? Which SNPs were exactly removed? 196 - (106 + 39) = 51 out of 196 removes almost three quarter of the genetic variants used as IVs. Is that reasonable?
It would be interesting to see scatterplots where SNPs that were removed are highlighted or color-coded.

Minor comments:

- Page 4, line 67-8: "case-control studies (11,221 cases and 68 16,168 controls)c found that" Remove c in)c.
- IVW: Specify if first or second order approximation of the variance was used.

Reviewer #3 (Remarks to the Author):

In this work, the authors present a Mendelian randomization analysis using GWAS results to evaluate the causal effect of smoking initiation and alcohol use on oral/oropharyngeal cancer. In addition, they perform multivariable Mendelian randomization to demonstrate evidence for an independent causal effect of smoking or drinking on oral/oropharyngeal cancer. The authors conduct a subtype analysis to evaluate potential heterogeneity in the causal effects. Lastly, other risky behaviors are analyzed for causal effects. There are novel aspects to the study that increase the potential impact, but there are also limitations in its current form that limit this impact. These are described below.

1) Multivariate MR estimates the direct causal effects of each exposure in a single analysis. Intuitively, this effect estimate is smaller than OR estimate in univariable MR. However, they are 2.49 (uni) vs. 3.6 (multi) for smoking; for alcohol use, they are 4.06 (uni) vs. 2.62 (multi), which makes more sense to me. Please explain how to interpret the numeric values.

2) For multivariable MR, please justify the validity of removing variables from the analysis. Has a pleiotropic association of such a variant been justified?
https://www.ncbi.nlm.nih.gov/pmc/articles/PMC7035387/

3) For the two-sample multivariable MR, one or more methods can be performed to show robustness of the primary findings. A recent work by Zuber et al. that uses multivariable MR can be considered.

4) why limit to risk tolerance and lifetime number of sexual partners for additional MR analysis? – Tobacco and alcohol use has been shown to be genetically correlated with many other factors. Moreover, smoking and drinking are thought to be risk factors of other factors. It may be of interest to see what the indirect effect of smoking/drinking on oral/oropharyngeal cancer by a dissection approach applied by Burgess et al. in here https://www.ncbi.nlm.nih.gov/pmc/articles/PMC5629317/.

5) The authors may discuss the potential biology behind the causal relationship they've claim. How smoking/drinking increases the risk of oral/oropharyngeal cancer? Please provide evidence from the literature in the discussion. Not necessarily the actual number, but how the causal effect of alcohol is modified by smoking and vice versa?

6) The format of figure 2, figure 3 and figure 4 (choice of color, legend, layout) needs improvement. They need to divide approaches more explicitly.

Response to referees

Please find enclosed, a revised version of our paper entitled “Investigating smoking and alcohol consumption in oral and oropharyngeal cancer: a multivariable Mendelian randomization analysis”.

We thank both reviewers for their comments and have responded to all points in order below. All revisions have also been highlighted in yellow within the revised manuscript.

Reviewer #1

- **Formalising smoking as exposure: Why did the authors select smoking initiation to formalise smoking exposure in the first instance? It is great to add the comprehensive smoking index later, but why did the authors present this as a separate paragraph and not show this in the main analysis?**

We initially selected smoking initiation and drinks per week as our main exposures of interest given that the largest genome-wide association studies (GWAS) for smoking and alcohol have been performed in relation to these traits in 1,232,091 and 941,280 individuals, respectively, as part of the GWAS and Sequencing Consortium of Alcohol and Nicotine (GSCAN). In addition, availability of GWAS summary statistics on subsets of the GSCAN data allowed us to evaluate the exposures in independent samples (i.e. GSCAN without UK Biobank and with UK Biobank only) and assess the robustness of effect estimates and also multivariable causal estimates. The GWAS for comprehensive smoking index (CSI) represents a smaller GWAS, conducted in 462,690 participants in UK Biobank.

We have now moved the investigation of comprehensive smoking index to the main analysis, as suggested by the reviewer. On further reflection, we believe this is justified given that the comprehensive smoking index serves as a quantitative measure of lifetime use, and therefore, is more comparable to the analysis of alcohol drinks per week. The manuscript has been updated in line with this.

- **What do the multivariable Mendelian randomization results look like when using data from the comprehensive smoking index?**

The multivariable Mendelian randomization (MR) analysis presented in the manuscript has now been performed in relation to the comprehensive smoking index (CSI) and alcohol drinks per week (DPW).

"In the multivariable MR analysis controlling for alcohol consumption, there was strong evidence for a direct causal effect of lifetime smoking behaviour on risk of oral/oropharyngeal cancer (IVW OR 2.6, 95% CI= 1.7, 3.9 per SD increase in the CSI). In multivariable MR analysis controlling for lifetime smoking, there was also strong evidence for a direct causal effect of alcohol consumption on risk of oral/oropharyngeal cancer (IVW OR 5.2, 95% CI= 3.2, 8.6 per SD increase in drinks per week)." (page 9, lines 163 – 168)

As described above, we agree that the comprehensive smoking index provides a more useful comparison to alcohol exposure than the binary variable for smoking initiation. For the multivariable MR analysis, the estimates used were obtained from different samples (i.e. comprehensive smoking index in UK Biobank and drinks per week in GSCAN without UK Biobank), in order to ensure that the pairwise covariances of the error in the estimated association between each SNP and each pair of exposures are zero. Findings from this analysis were similar to those performed previously in relation to smoking initiation and alcohol intake frequency, where an independent effect of both smoking and alcohol was found in multivariable MR. However, in the updated analysis, the interpretation and

comparison of effects is now clearer (representing the odds of head and neck cancer per SD increase in both lifetime smoke exposure and drinks per week).

The updated analysis also improves upon the previous multivariable MR investigation, which used alcohol intake frequency in UK Biobank rather than drinks per week. The reasoning for this is threefold:

1) The genetic instruments used were derived in relation to drinks per week rather than alcohol intake frequency and so it is more appropriate to use these in MR with drinks per week exposure.

2) The genetic contribution to drinks per week and alcohol intake frequency were recently found to show opposite genetic correlations with downstream health outcomes[1], suggesting that they shouldn't be used interchangeably.

3) The MR analysis assumes a dose-response in risk of head and neck cancer, with respect to the exposure variable. While this is reasonable for the quantity of alcohol consumed in drinks per week, the interpretation for alcohol intake frequency is not as straightforward as it assumes a consistent difference in risk between categories (never drinking, special occasions, 1-3 times a month, 1-2 times a week, 3-4 times a week, daily).

- **Risky behaviour: The authors also perform a univariable MR study where they look at the effect of other risky behaviours (risk tolerance and lifetime number of sexual partners) on the outcome. It would be nice to perform a multivariable MR study with**

both smoking, alcohol and risky behaviour to investigate attenuation when accounting for risky behaviour.

In assessing conditional instrument strength for alcohol and smoking in the multivariable MR analysis, we found that the genetic instruments were particularly weak in relation to drinks per week (F-statistic 6.79 for 60 SNPs and 4.94 for 59 SNPs, excluding *ADH1B*) (**Table 3**). Adding in a third variable into the multivariable MR analysis (such as risky behaviour) would reduce the strength of the conditional instrument even further which could reduce power to detect effects and bias estimates. Given this, and the fact that we found limited evidence for a strong causal effect of risk tolerance and lifetime number of sexual partners on overall head and neck cancer risk (**Figure 4**), we do not believe that the suggested analysis would contribute to the interpretation of the results and therefore did not include it.

- **In the discussion the authors say: "unlikely to be strongly influenced by other risky behaviours" this needs to be rephrased as only two types of risky behaviours (risk tolerance and lifetime number of sexual partners) were actually investigated.**

The phrase "unlikely to be strongly influenced by other risky behaviours" has now been changed to "*unlikely to be strongly influenced by risk tolerance and lifetime number of sexual partners*" *(page 10, lines 203 – 204)* to reflect that only two risky behaviours were investigated.

- **"There was no direct overlap between the SNPs used to instrument alcohol consumption and smoking initiation." What exactly do you mean by this? What do we learn from this? SNPs may be in LD ... Please clarify or delete this sentence.**

The issue of genetic correlation amongst the instruments used in this study has now been expanded upon and clarified in the Methods section:

"Linkage disequilibrium statistics (r^2) were evaluated to check the overlap between smoking initiation, comprehensive smoking index and drinks per week loci using LDmatrix. All pairwise correlations were low for the SNPs used to instrument smoking initiation and drinks per week (r^2 <0.06) and those used to instrument comprehensive smoking index and drinks per week (r^2 <0.07), with the exception of rs10236149 and rs6962772 which had an r^2 of 0.743. For smoking initiation and comprehensive smoking index, there were 6 duplicate SNPs and 11 SNP pairs which were in strong LD (r^2 >0.8)." (page 17-18, lines 354 – 360).

- **To account for directional pleiotropy, we compared results with three other MR methods, which each make different assumptions about this: MR-Egger, weighted median and weighted mode. Do median and mode account for directional pleiotropy? Both methods are robust to general pleiotropy but NOT directional pleiotropy. You may want to rephrase: "To account for pleiotropy".**

We agree that weighted median and mode are robust to general, not directional, pleiotropy and have rephrased *"To account for pleiotropy" (page 18, line 378)*, deleting the word directional.

- **So far the manuscript only presents scatter and leave one out plots for alcohol as exposure. Please include into the Supplementary, similar plots for all analysis (Smoking as exposure in univariable MR and multivariable MR).**

Additional scatter and leave one out plots have been added for smoking initiation and the comprehensive smoking index for completeness (**Supplementary Figure 1**).

- **The outlier selection for multivariable MR needs more details. How was the Q-statistic computed? Which SNPs were exactly removed? 196 - (106 + 39) = 51 out of 196 removes almost three quarter of the genetic variants used as IVs. Is that reasonable? It would be interesting to see scatterplots where SNPs that were removed are highlighted or color-coded.**

The multivariable MR analysis now presented based on the comprehensive smoking index and alcohol drinks per week, revealed limited evidence for heterogeneity based on the Q-statistic, estimated using the "MVMR" package in R [2]. As such, the analysis removing genetic variants, further to the main *ADH1B* outlier, to reduce heterogeneity is no longer relevant and has not been included (**Table 3**).

- **Minor comments:**

  Page 4, line 67-8: "case-control studies (11,221 cases and 16,168 controls)c found that…"

We have removed c in *)c (page 4, line 67)*.

IVW: Specify if first or second order approximation of the variance was used.

"First-order inverse-variance weights were used to calculate both the IVW estimate and Cochran's Q. A random effects model was used in the presence of heterogeneity [3]."

This has been updated in both the main manuscript *(page 18, line 373 – 374)* and glossary *(page 22, line 464 – 466).*

Reviewer #3

- **Multivariate MR estimates the direct causal effects of each exposure in a single analysis. Intuitively, this effect estimate is smaller than OR estimate in univariable MR. However, they are 2.49 (uni) vs. 3.6 (multi) for smoking; for alcohol use, they are 4.06 (uni) vs. 2.62 (multi), which makes more sense to me. Please explain how to interpret the numeric values.**

The multivariable MR analysis presented in the revised paper uses the comprehensive smoking index (CSI) and drinks per week (DPW). In agreement with reviewer #1, we believe that the comprehensive smoking index provides a more useful comparison to the continuous measure of alcohol exposure (i.e. drinks per week), than the binary variable for smoking initiation (SI) and serves as a more accurate quantitative measure of lifetime use. In the updated multivariable MR analysis, the estimates obtained for both smoking and alcohol were consistently smaller than the equivalent univariable MR estimates (**Figure 3**).

- **For multivariable MR, please justify the validity of removing variables from the analysis. Has a pleiotropic association of such a variant been justified?**

The multivariable MR analysis now presented based on the comprehensive smoking index and drinks per week, revealed limited evidence for heterogeneity based on the Q-statistic, estimated using the “MVMR” package in R. As such, the analysis removing genetic variants, further to the main *ADH1B* outlier, to reduce heterogeneity is no longer relevant and has not been included (**Table 3**). We believe that the exclusion of the *ADH1B* variant is justified

since it was determined to be an influential outlier in MR-PRESSO and removal of this variant improves both the MR Egger intercept estimate and reduces heterogeneity in both univariable and multivariable MR. This indicates potential pleiotropy of the variant and its pleiotropic role with respect to head and neck cancer has been described.

"Our finding that the ADH1B variant, rs1229984 was a clear outlier which inflated causal estimates, indicates pleiotropy of this variant via its role in alcohol metabolism. However, we found consistent evidence for a causal effect of alcohol on oral and oropharyngeal cancer risk when this variant was excluded from the analysis." (page 14, lines 284 – 287)

- **For the two-sample multivariable MR, one or more methods can be performed to show robustness of the primary findings. A recent work by Zuber et al. that uses multivariable MR can be considered.**

MR-BMA is a two-sample multivariable MR approach based on Bayesian model averaging, which can be used to analyse a high-dimensional set of risk factors. It does however use a standard multivariable MR IVW estimation, the same method applied in this paper. As stated in the Zuber et al. paper (2020), the primary aim of their method is to detect causal risk factors (by searching possible combinations of exposures) rather than to unbiasedly estimate the magnitude of causal effects. We do not believe it is a suitable method for application in the current MR study, which is focused on estimating the magnitude of known risk factors (smoking and alcohol) for head and neck cancer, rather than a hypothesis-free exploration of novel or less well established risk factors.

- **Why limit to risk tolerance and lifetime number of sexual partners for additional MR analysis? – Tobacco and alcohol use has been shown to be genetically correlated with many other factors. Moreover, smoking and drinking are thought to be risk factors of other factors. It may be of interest to see what the indirect effect of smoking/drinking on oral/oropharyngeal cancer by a dissection approach applied by Burgess et al. in here https://www.ncbi.nlm.nih.gov/pmc/articles/PMC5629317/.**

We acknowledge the reviewer's comment to consider further exploration of other risky behaviours. We will justify why only alcohol, smoking, lifetime sexual behaviour and risk tolerance were selected.

The main aim of this paper was to re-estimate the independent effects of smoking and alcohol on head and neck cancer risk, rather than to identify novel risk factors. The rationale for focusing on alcohol and smoking is supported by the largest pooled analysis[4] from 17 European and American head and neck cancer case-control studies (11,221 cases and 16,168 controls) which found that, of the population attributable risk of smoking and alcohol, 4% could be attributed to alcohol alone and 33% to tobacco alone, with 35% explained by a greater than multiplicative joint effect of alcohol and tobacco combined for oral and oropharyngeal cancer. Further evidence to support the direct carcinogenic mechanisms of smoking and alcohol have now been included in the paper (page 12, lines 234– 242). While these risk factors are well known, the independent contributions of both smoking and alcohol on head and neck cancer are not clear, since these behaviours tend to co-occur. This is what our study attempts to address.

The MR methods applied here, attempt to determine the causal and independent role of both lifetime smoking and alcohol consumption on head and neck cancer risk. We performed a sensitivity analysis to consider the role of the human papilloma virus (HPV), a third well-established risk in oropharyngeal cancer [5]. The high-risk HPV subtypes found in the mouth are almost entirely sexually transmitted, so oral sex is likely the primary route of transmission. As this phenotype isn't available in current datasets, lifetime number of sexual partners and general risk tolerance were used as proxy measures. A pooled analysis has demonstrated that this is an effective measure for capturing HPV transmission, where a history of six or more lifetime sexual partners increased the risk of oropharyngeal cancer (OR 1.25, 95%CI 1.01, 1.54) [4]. Further justification for this sensitivity analysis is now provided in the introduction, with appropriate references:

"Finally, given evidence of strong genetic correlation for both smoking and alcohol intake with other risky behaviours [6], and the established link between sexual behaviour and HPV-driven head and neck cancer [5], we conducted additional MR analysis of both risk tolerance and lifetime number of sexual partners on oral cavity (OC) and oropharyngeal cancer (OPC)." (page 5, lines 88 – 92)

While we agree that it is of interest to apply MR to consider the causal role of other less well-established risk factors for head and neck cancer, that was not the goal of this study.

- **The authors may discuss the potential biology behind the causal relationship they've claim. How smoking/drinking increases the risk of oral/oropharyngeal cancer? Please provide evidence from the literature in the discussion. Not necessarily the actual number, but how the causal effect of alcohol is modified by smoking and vice versa?**

The following section discussing potential biological mechanisms has been added to the Discussion section, with supporting literature (see updated References):

"Several mechanisms have been suggested to explain the observed associations between alcohol and risk of head and neck cancer, including contaminants present in alcoholic drinks (e.g. N-nitrosodiethylamine in beer) [7], free radical damage and impairment of DNA repair capacity [8,9]. Ethanol is oxidised to acetaldehyde, which has a direct carcinogenic effect and moreover alcohol may act as a 'solvent' for tobacco carcinogens and the induction of carcinogen-metabolising enzymes [10,11]. Given their strong co-existence, the effects of smoking and alcohol are often difficult to separate out. However, in addition to the reported synergistic effect, previous observational data has highlighted an independent effect of both agents [12], with our results supporting this." (page 12, lines 234– 242).

- **The format of figure 2, figure 3 and figure 4 (choice of color, legend, layout) needs improvement. They need to divide approaches more explicitly.**

New **Figures 2, 3** and **4** have been generated, with clear borders to separate the different approaches. The colours, legend and layout has also been improved.

- **Other changes made:**

Data and code availability statements have now been added:

Data availability statement

"*GWAS summary statistics for the SNPs used to estimate the causal effects of smoking initiation, lifetime smoking and alcoholic drinks per week are presented in* ***Supplementary Tables 1*** *and* ***5****. Full summary statistics for the GAME-ON GWAS can be accessed via dbGAP (OncoArray: Oral and Pharynx Cancer; study accession number: phs001202.v1.p1) [13].*

Code availability statement

Two-sample MR analyses were conducted using the "TwoSampleMR" package in R (version 3.5.3). Multivariable MR analysis was conducted using both the "MVMR" and "MendelianRandomization" [14] packages in R. A copy of the code used in this analysis is available at https://github.com/rcrichmond/smoking_alcohol_headandneckcancer

Detail on a recently published MR study in UK Biobank [15] has been added to the discussion:

"A more recent multivariable MR study in UK Biobank [15] investigated smoking initiation and alcohol consumption with multiple cancers. This study demonstrated that smoking initiation increased odds of head and neck cancer when adjusted for alcohol (IVW OR 1.4, 95% CI= 1.1, 1.8, p=0.002). There was also a strong positive effect of alcohol consumption on head and neck cancer risk when adjusted for smoking (IVW OR 1.9, 95% CI= 0.8, 4.6, p= 0.142), although with wider confidence intervals [15]." (page 13 lines 257 – 263).

Updated References

1 Marees, A. T. *et al.* Potential influence of socioeconomic status on genetic correlations between alcohol consumption measures and mental health. *Psychol Med* **50**, 484-498, doi:10.1017/S0033291719000357 (2020).

2 Sanderson, E., Spiller, W. & Bowden, J. Testing and Correcting for Weak and Pleiotropic Instruments in Two-Sample Multivariable Mendelian Randomisation. *bioRxiv*, 2020.2004.2002.021980, doi:10.1101/2020.04.02.021980 (2020).

3 Bowden, J. *et al.* Improving the visualization, interpretation and analysis of two-sample summary data Mendelian randomization via the Radial plot and Radial regression. *Int J Epidemiol* **47**, 1264-1278, doi:10.1093/ije/dyy101 (2018).

4 Hashibe, M. *et al.* Interaction between Tobacco and Alcohol Use and the Risk of Head and Neck Cancer: Pooled Analysis in the International Head and Neck Cancer Epidemiology Consortium. *Cancer Epidem Biomar* **18**, 541-550, doi:10.1158/1055-9965.Epi-08-0347 (2009).

5 Gillison, M. L., Chaturvedi, A. K., Anderson, W. F. & Fakhry, C. Epidemiology of Human Papillomavirus-Positive Head and Neck Squamous Cell Carcinoma. *J Clin Oncol* **33**, 3235-3242, doi:10.1200/JCO.2015.61.6995 (2015).

6 Karlsson Linner, R. *et al.* Genome-wide association analyses of risk tolerance and risky behaviors in over 1 million individuals identify hundreds of loci and shared genetic influences. *Nat Genet* **51**, 245-257, doi:10.1038/s41588-018-0309-3 (2019).

7 Rogers, M. A., Vaughan, T. L., Davis, S. & Thomas, D. B. Consumption of nitrate, nitrite, and nitrosodimethylamine and the risk of upper aerodigestive tract cancer. *Cancer Epidemiol Biomarkers Prev* **4**, 29-36 (1995).

8 Wight, A. J. & Ogden, G. R. Possible mechanisms by which alcohol may influence the development of oral cancer--a review. *Oral Oncol* **34**, 441-447, doi:10.1016/s1368-8375(98)00022-0 (1998).

9 Olshan, A. F., Weissler, M. C., Watson, M. A. & Bell, D. A. Risk of head and neck cancer and the alcohol dehydrogenase 3 genotype. *Carcinogenesis* **22**, 57-61, doi:10.1093/carcin/22.1.57 (2001).

10 Dellarco, V. L. A mutagenicity assessment of acetaldehyde. *Mutat Res* **195**, 1-20, doi:10.1016/0165-1110(88)90013-9 (1988).

11 Homann, N. *et al.* Effects of acetaldehyde on cell regeneration and differentiation of the upper gastrointestinal tract mucosa. *J Natl Cancer Inst* **89**, 1692-1697, doi:10.1093/jnci/89.22.1692 (1997).

12 Rothman, K. & Keller, A. The effect of joint exposure to alcohol and tobacco on risk of cancer of the mouth and pharynx. *J Chronic Dis* **25**, 711-716, doi:10.1016/0021-9681(72)90006-9 (1972).

13 Lesseur, C. *et al.* Genome-wide association analyses identify new susceptibility loci for oral cavity and pharyngeal cancer. *Nat Genet* **48**, 1544-1550, doi:10.1038/ng.3685 (2016).

14 Yavorska, O. O. & Burgess, S. MendelianRandomization: an R package for performing Mendelian randomization analyses using summarized data. *Int J Epidemiol* **46**, 1734-1739, doi:10.1093/ije/dyx034 (2017).

15 Larsson, S. C. *et al.* Smoking, alcohol consumption, and cancer: A mendelian randomisation study in UK Biobank and international genetic consortia participants. *PLOS Medicine* **17**, e1003178, doi:10.1371/journal.pmed.1003178 (2020).

Reviewer #1 (Remarks to the Author):

The authors have addressed all of my comments. I would like to congratulate them for their manuscript and the interesting and comprehensive MR analysis to understand risk factors of oral and oropharyngeal cancer.

Reviewer #3 (Remarks to the Author):

The authors have sufficiently responded to all of my comments.

Response to referees

We would again like to thank the reviewers for their final comments.

All reviewer comments have been addressed in the previous revision, so no further changes were made.

Reviewer #1 (Remarks to the Author):

The authors have addressed all of my comments. I would like to congratulate them for their manuscript and the interesting and comprehensive MR analysis to understand risk factors of oral and oropharyngeal cancer.

Reviewer #3 (Remarks to the Author):

The authors have sufficiently responded to all of my comments.
